# Socioeconomic correlates of Adequate Maternal Care in Bangladesh: Analysis of the Bangladesh Demographic and Health Survey 2017-18

**DOI:** 10.1155/2022/8027712

**Published:** 2022-11-08

**Authors:** Md. Injamul Haq Methun, Iqramul Haq, M. Sheikh Giash Uddin, Azizur Rahman, Saiful Islam, Md. Ismail Hossain, Shatabdi Shamrita Ume, Md. Jakaria Habib, Sutopa Roy

**Affiliations:** ^1^Department of Statistics, Tejgaon College, Dhaka, 1215, Bangladesh; ^2^Department of Agricultural Statistics, Sher-e-Bangla Agricultural University, Dhaka, 1207, Bangladesh; ^3^Department of Statistics, Jagannath University, Dhaka, 1100, Bangladesh; ^4^Department of Statistics, Jahangirnagar University, Dhaka, Bangladesh; ^5^Department of Statistics, Shibalaya Sadar Uddin Degree College, Manikganj-1850, Bangladesh

## Abstract

**Introduction:**

In recent times, Bangladesh has made significant improvements in various health outcomes, but not so much in maternal death. The current flat trend in reducing maternal mortality in Bangladesh has been mainly due to the lower coverage of maternal health care. To improve the coverage, it is essential to find biosocial factors related to adequate maternal health care. Therefore, this study is aimed at finding out the socioeconomic correlates of adequate maternal health care in Bangladesh.

**Methods:**

The study used data from the Bangladeshi demographic and health survey 2017-18. The total unweighted sample of 4012 women who reported pregnancy before three years of the survey was analyzed. A composite binary indicator of adequate maternal care has been constructed using the variables—access to maternal care service, four antenatal care visits, at least one visit with qualified providers, and institutional delivery. A binary logistic regression model was employed to find out the socioeconomic correlate of adequate maternal care.

**Results:**

Only 24.4% percent of sample women received adequate maternal care. The result of the logistic regression model shows that urban, Khulna, Rajshahi, and Rangpur were associated with an increase in the odds of having adequate maternal care. High education and health care decisions taken by the partner or husband were also associated with an increased odd of adequate maternal care. Islam and lower wealth status were associated with a lower probability of adequate maternal care.

**Conclusions:**

Policymakers and health administration should pay attention to the variation in the utilization of maternal health care across residence, region, religion, education, and wealth status to ensure safe motherhood.

## 1. Introduction

In 2017, almost 295,000 mothers died around the world due to pregnancy-related causes [1]. Most of these maternal deaths occurred in sub-Saharan Africa and South Asia. The maternal mortality rate in sub-Saharan Africa remains alarmingly high, even though it has fallen by 40% since 1990 [2]. Though maternal death in South Asia has declined substantially (59%) since 2000, this region alone contributed 19% to global maternal deaths [3]. Maternal health status in Bangladesh remains a significant concern [4]. The maternal mortality ratio (MMR) was estimated at 163 per 100,000 live births in 2020, which was higher than other south-Asian countries India 145, Pakistan 140, and Sri Lanka 36 [3, 5]. Bangladesh recently made tremendous progress toward achieving the Sustainable Development Goal (SDG) target of goal 3 and is very close to achieving neonatal mortality rate (NMR) = 12 and under 5 mortality ratios (U5MR) = 25 per 1,000 live births [6]. However, with significant maternal deaths, Bangladesh remains far behind in achieving another target of SDG Goal 3, the maternal mortality ratio of 70 per 100,000 live births. In developing nations, delivery and pregnancy-related complications were leading causes of maternal mortality among 15-49 year old women [2]. Lower middle and low-income countries accounted for 99% of preventable maternal death [7]. Due to inadequate access to sufficient prenatal care, millions of mothers in developing and under-developed nations are often encountering life-threatening pregnancy-related difficulties [8].

Increased usage and access to key essential maternal health care services, such as antenatal care (ANC), institutional delivery, and postnatal care, can prevent millions of maternal deaths [9]. World Health Organization (WHO) has recommended four antenatal visits, one in the first trimester of pregnancy as part of adequate care indicators [8]. ANC is a key primary program designed to protect and foster healthy mothers and healthy babies by early detection of disease in pregnant women and providing the appropriate treatment [2, 10]. In addition, it ensures delivery in a well-equipped facility for women at high risk of complicated delivery by timely identification [2]. Skilled health personnel are also essential for identifying and mitigating risk factors in pregnancy [8]. ANC has provided significant achievement for maternal health in the developed nations for a long time. However, in middle- and low-income countries, most of the ANC initiatives have provided unsatisfactory results. In the regions where most countries are developed, such as the Caribbean and European regions, 90% of women make at least four ANC visits. Whereas, in developing countries, coverage of at least four ANC visits is low, 49% in South Asia [11]. In Bangladesh, only 47% of women have undergone four ANC visits and 82% of women receive ANC from a medically trained provider [12]. The presence of skilled delivery attendants is also an important intervention for maternal and child health outcomes [3]. Only 53% of deliveries in Bangladesh were performed by medical personnel [12].

The issue of adequate maternal care continues to pique the interest of researchers. Several studies have shown that providing adequate maternal health care can lower maternal death rates. According to Srivastava et al. [13], maternal health care satisfaction is influenced by a number of structural, process, and outcome characteristics. According to Sanogo and Yaya [14], women who enroll in health insurance programs and improve their household's financial situation can increase the efficiency of health service consumption, resulting in more women receiving adequate maternal health care. Gebrekirstos et al. [8] found that tertiary and higher education, support from the spouse, a high-income index, and follow-up in private facilities, among other factors, were essential drivers of acceptable use of antenatal care services. These findings are significant in terms of maternal health care service consumption. Adequate maternal health care, on the other hand, is not just dependent on the use of health services; socioeconomic factors may also influence it. We look at these socioeconomic characteristics in this paper to see if they may be used as predictors of sufficient maternal health care in the context of Bangladesh.

In this document, the socioeconomic factors that affect sufficient maternal health care in Bangladesh were identified.

## 2. Methodology

### 2.1. Data Source

This study organized data from Bangladesh Demographic and Health Survey 2017-18 dataset. It is a nationally representative cross-sectional survey. Collect demographic and health-related information every 3 to 4 years. The Demographic and Health Survey (DHS) has been carried out in Bangladesh since 1993 [12].

### 2.2. Sampling design and sample size

This survey was conducted based on two-stage stratified cluster sampling technique. Only the women of reproductive age (15 to 49 years) were considered as respondents.

After getting the permission by online registration, the SPSS version 20.0 of the data was downloaded from DHS. Unweighted women data from Bangladesh's demographic and health survey 2017-18 was used for this analysis. Among the women, only 5,012 women were reported the pregnancy in three years preceding the survey. Thus, the study population was formed through these 5,012 women.

### 2.3. Study variables

We consider access to the antenatal care service, 4 antenatal care visits, at least one visit with qualified providers, and institutional delivery as indicators of adequate maternal health care. Using these variables, a composite indicator of adequate maternal health care was constructed. The composite indicator has value 1 when all of the following four conditions are satisfied by a woman: had access to ANC, received at least four ANC visits, at least one visit with qualified doctors, and had institutional delivery. The composite indicator also takes a value 0 if any of the above conditions were not satisfied by a woman. Based on previous literature, age, residence, division, education, sex of the household head, wealth status, religion, working status, health care decision-maker, and age at birth were taken as independent variables.

### 2.4. Statistical analysis

Descriptive analysis was conducted to demonstrate the background characteristics of the respondents. Then, chi-square test statistics were used to investigate the association between sociodemographic variables and adequate maternal health care. In further analysis, the logistic regression model has been used to estimate the effects of sociodemographic predictors on adequate maternal health care.

All statistical analyses were conducted using R (4.0.3).

## 3. Results

Most of the mothers live in rural areas (65.6%). The percentage of mothers is the highest in the 20 to 24 age group (35.4%); mothers with secondary education (53.1%); mothers living in a household with a male household head (87.9%); mothers with no working status (62.5%); mothers who had institutional delivery (50.3%); mothers who had access to ANC care (91.9%); mothers who had access to a qualified provider (83.2%); mothers who did not complete four ANC visits (64%); mothers with no adequate care (75.6%); mothers living in household where health care decisions were taken by husband (64.6%); and mothers with age at childbirth was between 20 to 34 (70.8%) (Table 1).

Table 2 clarified the percentage distribution of adequate maternal health care, which was accompanied by sociodemographic characteristics in Bangladesh. It also represented the factors that significantly associated (*p* < 0.001) with adequate care, including residence, division, education, wealth status, working status, and health care decisions.

The residence of mothers is significantly interconnected with adequate care of mothers in Bangladesh. Almost 36.46% of mothers from urban areas received adequate care and only half (18.13%) in rural areas.

Mothers from the Dhaka (31.71%) and Khulna (31.11%) divisions received the most adequate health care than the other divisions. Less than 20% of mothers from Barisal (19.89%), Mymensingh (18.74%), and Sylhet (18.26%) divisions received adequate care. In Table 2, the data reveals a significant positive association between education and adequate health care. It was prominent that 47.27% of mother with higher education received adequate care while 21.14% of mothers with secondary, 10.06% with primary and only 6.73% of mother with no education received adequate care.

More than half (51.12%) of the richest families, 28.64% of the richer families, and 22.54% of the middle-class families got adequate care. Among the poorer and poorest mothers, 12.09% and 8.53% received adequate care, respectively. Non-Muslim women had more adequate health care facilities than Muslim women (29.31% vs. 29.99%). Furthermore, the adequate health care was lower among working mothers (20.11%) than for nonworking mothers (27.04%). In terms of health care decisions, women who were taking health care decisions with their husbands had more adequate health care (26%) (Table 2).

A binary logistic regression model was used to determine factors related to adequate maternal health care in Bangladesh, and the results are shown in Table 3. A significant association was found between residence and adequate care. When compared to rural women, urban women had a 1.42 times higher likelihood of receiving adequate care (OR = 1.421, 95% CI = 1.213–1.672). The results indicate that mothers from Dhaka (OR = 1.391), Khulna (OR = 1.794), Rajshahi (OR = 1.869), and Rangpur (OR = 2.142) divisions had significantly higher odds of receiving adequate health care than women from the Sylhet division.

There was a positive association between the mother's educational level and adequate care, as shown in Table 3. The highest odds of receiving adequate health care occurred among mothers with higher education, compared to women who had no formal education. Mothers with secondary education and higher education were 2.74 times and 5.57 times, respectively, more likely to receive adequate health care than mothers with no education.

Additionally, a positive association was also found between wealth status and adequate care. Mothers from richer, middle, poorer, and poorest families were less likely to have adequate health care, respectively, than women from the richest families. Mothers who practiced Islam had a lower chance of adequate care (OR = 0.751, 95% CI = 0.586–0.983) than mothers from other religions. Women whose health care decisions were made by their husbands were 1.227 times more likely to have adequate care compared to the women whose health care decisions were made by other people.

## 4. Discussion

Maternal nutritional status is the result of the relationship between food consumption, health status, and access to health care facilities [15]. Poor maternal care is one of the most important health and welfare problems faced by Bangladeshi women. So, the studies of determinants of maternal care provide fruitful suggestions to the health care authorities, providers, and planners of the public health sector of a country's government; specifically for a country like Bangladesh [16]. In Bangladesh, majority of women do not get any health service; they deliver their children at home with unskilled or nonprofessional attendants [17]. According to a study, health facility delivery, education of the respondent, education of the partner, residence area, division, religion, wealth index, and age at first birth were significant variables in the use of maternal health care among Bangladeshi women [17]. A similar study conducted by Banik in Rajshahi said that distance appears to work as a key deterrent factor in accessing adequate health facilities [18].

The current study includes socioeconomic and variables. The strength of this study is that it has a large national representative data set. For statistical benefits, both bivariate and multivariate techniques have been applied in this study. Maternal care was associated with individual-level factors such as residence, division, age, and religion [16, 18]. Several other individual and household factors including educational level of women and household wealth status were inversely associated with it. The significance remained the same for other additions of community-level factors, such as health care decision-making [15, 16].

In developing countries such as Bangladesh, regional variance is a common factor in various public health indicators such as maternal health care; the existing study also shows similar results[15]. Women who belong to rural areas are less likely to be underweight and do not get adequate health care compared to their counterparts in urban areas [19]. Previous researchers have supported the presence of inequalities in the use of institutional delivery facilities at all income and education levels [20]. Divisional disparity is high in Bangladesh as access to adequate healthcare is less in the northern part of the northern part of the country, like in Rangpur and Rajshahi [18]. A special campaign needed for the development of current scenario in rural areas and specific districts. Rural mothers should be encouraged to seek healthcare in government facilities and NGO's [15, 18].

Our findings suggest that socioeconomic variables are important determinants of adequate health care are similar to those of earlier studies examining these associations in Bangladesh. Household wealth status is a well-furnished determinant of maternal health care. Like ours, previous studies also showed that mothers in households with poor socioeconomic status experience a greater risk of being underweight than those with high socioeconomic status [15]. About half (48%) of South Asian people are multidimensionally poor, as a result, social and economic factors could impact the ANC visits of South Asian women in various ways [21].

Other studies suggest that the exposure to religious thoughts or practices, could shape women's decision-making about reproductive health care and related practices [21]. From that point of view, religion remains one of the significant tools that could be explored to increase the utilization of health facilities among them. These problems arose from a number of specific factors including, a religious restriction to maintain modest dressing and the ignorance of unlawful bodily exposure or contact with specific people including male caregivers, lack of personal privacy in healthcare facilities [21, 22].

Our findings also agreed with previous study that both working women and literacy are related to adequate maternal care [23]. Furthermore, none or primary levels of education can be associated with a lack of adequate health care compared to mothers who have higher education. Education also enhances the autonomy of mothers to make decisions and the capability to use proper quality services that offer better health care [24]. Studies show that there is a strong relationship between women's decision-making autonomy and the use of ANC care during pregnancy and PNC within 2 days after delivery, even after adjusting for sociodemographic variables in South Asian regions such as Bangladesh [24].

Our findings showed that child marriage hinders the access of mothers to adequate maternal care. It is important for women to know when to have children, the associated risk factors and complications during of labor and childbirth. Therefore, women who are married at an earlier age may have an increased risk of maternal mortality and morbidity, as their problems and complications during pregnancy and delivery are less likely to be removed and treated in a timely manner [25].

## 5. Conclusions

In summary, we conclude that residence, division, education, wealth status, religion, and health care decision taken by husband were potential risk factors for adequate care of mothers in Bangladesh. Improving maternal health is a worldwide public health importance. This study demonstrates the access and use of maternal health care in Bangladesh, which is influenced by various socioeconomic characteristics of pregnant women. The results can be used to develop follow-up and assessment programs for pregnancy health care for pregnancy.

## Figures and Tables

**Table 1:** Univariate analysis.

Variables	Frequency	Percentage
Age		
15-19	869	17.3
20-24	1773	35.4
24-29	1310	26.1
30-34	749	14.9
35-49	311	6.2
Residence		
Urban	1725	34.4
Rural	3287	65.6
Division		
Barisal	533	10.6
Chattogram	835	16.7
Dhaka	741	14.8
Khulna	524	10.5
Mymensingh	603	12.0
Rajshahi	527	10.5
Rangpur	559	11.2
Sylhet	690	13.8
Education		
No education	312	6.2
Primary education	865	17.3
Secondary education	2663	53.1
Higher education	1172	23.4
Sex of Household Head		
Male	4404	87.9
Female	608	12.1
Wealth status		
Poorest	1079	21.5
Poorer	1017	20.3
Middle	905	18.1
Richer	988	19.7
Richest	1023	20.4
Religion		
Islam	4589	91.6
Other	423	8.4
Working status		
No	3132	62.5
Yes	1880	37.5
Sex of child		
Male	2624	52.4
Female	2388	47.6
Institutional delivery		
No	2492	49.7
Yes	2520	50.3
Access ANC care		
No	408	8.1
Yes	4604	91.9
Qualified provider		
No	844	16.8
Yes	4168	83.2
4 ANC visits		
No	3210	64.0
Yes	1802	36.0
Adequate maternal health care		
No	3787	75.6
Yes	1225	24.4
Health care decision		
Self	374	7.5
Partner/Husband	3236	64.6
Others	1402	28.0
Age at Child birth		
Less than 20	1239	24.7
20-34	3550	70.8
Greater than 34	223	4.4

**Table 2:** Bivariate analysis.

Variables	Adequate maternal health care		*p* value
	No (%)	Yes (%)	
Age			
15-19	78.14	21.86	0.08
20-24	76.25	23.75	
24-29	73.36	26.64	
30-34	74.10	25.90	
35-49	77.17	22.83	
Residence			
Urban	63.54	36.46	<0.001
Rural	81.87	18.13	
Division			
Barisal	80.11	19.89	<0.001
Chattogram	78.08	21.92	
Dhaka	68.29	31.71	
Khulna	68.89	31.11	
Mymensingh	81.26	18.74	
Rajshahi	72.11	27.89	
Rangpur	72.81	27.19	
Sylhet	81.74	18.26	
Education			
No education	93.27	6.73	<0.001
Primary education	89.94	10.06	
Secondary education	78.86	21.14	
Higher education	52.73	47.27	
Sex of Household Head			
Male	75.54	24.46	0.99
Female	75.66	24.34	
Wealth status			
Poorest	91.47	8.53	<0.001
Poorer	87.91	12.09	
Middle	77.46	22.54	
Richer	71.36	28.64	
Richest	48.88	51.12	
Religion			
Islam	76.01	23.99	0.01
Other	70.69	29.31	
Working status			
No	72.96	27.04	<0.001
Yes	79.89	20.11	
Sex of child			
Male	74.81	25.19	0.21
Female	76.38	23.62	
Health care decision			
Self	74.87	25.13	0.002
Partner/Husband	74.20	25.80	
Others	78.89	21.11	
Age at birth.			
Less than 20	78.53	21.47	0.01
20-34	74.39	25.61	
Greater than 34	77.58	22.42	

**Table 3:** Multivariate logistic regression analysis.

Variables	OR	95% CI		*p* value
		Lower	Upper	
Residence				
Urban	1.424	1.213	1.672	<0.001
Rural (ref.)	1			
Division				
Barisal	1.282	0.93	1.767	0.130
Chattogram	0.997	0.755	1.317	0.981
Dhaka	1.391	1.056	1.832	0.019
Khulna	1.794	1.331	2.418	<0.001
Mymensingh	1.305	0.952	1.789	0.098
Rajshahi	1.869	1.379	2.532	<0.001
Rangpur	2.142	1.573	2.916	<0.001
Sylhet (ref.)	1			
Education				
No education (ref.)	1			
Primary education	1.421	0.854	2.365	0.176
Secondary education	2.737	1.709	4.384	<0.001
Higher education	5.571	3.437	9.029	<0.001
Wealth status				
Poorest	0.170	0.127	0.228	<0.001
Poorer	0.218	0.167	0.285	<0.001
Middle	0.414	0.329	0.52	<0.001
Richer	0.491	0.402	0.601	<0.001
Richest	1			
Religion				
Islam	0.751	0.586	0.963	0.02
Other	1			
Working status				
No	1.124	0.959	1.318	0.15
Yes	1			
Health care decision				
Self	1.180	0.879	1.584	0.269
Partner/Husband	1.227	1.039	1.451	0.016
Others (ref.)	1			
Age at Child birth				
Less than 20	0.986	0.66	1.473	0.946
20-34	1.046	0.717	1.526	0.816
Greater than 34 (ref.)	1			

## Data Availability

In this study, we used data from the Bangladesh Demographic Health Survey (BDHS), 2017-18, which is available from https://dhsprogram.com/data/available-datasets.cfm.
